# Smooth enlargement of human standing sway by instability due to weak reaction floor and noise

**DOI:** 10.1098/rsos.150570

**Published:** 2016-01-06

**Authors:** Tetsuro Funato, Shinya Aoi, Nozomi Tomita, Kazuo Tsuchiya

**Affiliations:** 1 Department of Mechanical Engineering and Intelligent Systems, The University of Electro-communications, 1-5-1 Chofugaoka, Chofu, Tokyo 182-8585, Japan; 2 Department of Aeronautics and Astronautics, Kyoto University, Kyoto daigaku-Katsura, Nishikyo-ku, Kyoto 615-8540, Japan; 3 Department of Mathematics, Kyoto University, Kitashirakawa Oiwakecho, Sakyo-ku, Kyoto 606-8502, Japan; 4 JST, CREST, 5 Sanbancho, Chiyoda-ku, Tokyo 102-0075, Japan

**Keywords:** posture control, dynamical model, Hopf bifurcation, noise

## Abstract

Human quiet standing is accompanied by body sway. The amplitude of this body sway is known to be larger than would be predicted from simple noise effects, and sway characteristics are changed by neurological disorders. This large sway is thought to arise from nonlinear control with prolonged periods of no control (intermittent control), and a nonlinear control system of this kind has been predicted to exhibit bifurcation. The presence of stability-dependent transition enables dynamic reaction that depends on the stability of the environment, and can explain the change in sway characteristics that accompanies some neurological disorders. This research analyses the characteristics of a system model that induces transition, and discusses whether human standing reflects such a mechanism. In mathematical analysis of system models, (intermittent control-like) nonlinear control with integral control is shown to exhibit Hopf bifurcation. Moreover, from the analytical solution of the system model with noise, noise is shown to work to smooth the enlargement of sway around the bifurcation point. This solution is compared with measured human standing sway on floors with different stabilities. By quantitatively comparing the control parameters between human observation and model prediction, enlargement of sway is shown to appear as predicted by the model analysis.

## 1. Introduction

Humans typically maintain a standing posture by swaying backward and forward by approximately 20 mm at a very low frequency (less than 1 Hz). Medical and motor control research shows that characteristics of this body sway reflect the neural mechanism of posture control in the following ways. (i) Body sway changes are inherent symptoms of certain neurological diseases; for example, the amplitude of sway decreases with Parkinson’s disease [1,2], and the complexity of sway increases with spinocerebellar ataxia [3]. (ii) Biological noise, such as haemodynamic noise, does not by itself adequately explain the amplitude of body sway; to do so requires some nonlinear neural mechanism [4,5].

One possible explanation for large-amplitude sway is that it is a result of stabilization to a stationary state with a prolonged period of no control [6–9]. A control procedure called intermittent control, in which control is exerted intermittently [6–9], replicates the features of human body sway and is robust against cognitive delay. This intermittent control model shows a limit cycle state [10] and a micro-chaos state [9]; and a simulation study of standing on an unstable floor (a wobble board) with a nonlinear control model predicted a bifurcation structure [11]. This bifurcation structure allows involuntary transition of sway state with destabilization of the stationary state. By allowing the initiation of cyclic motion rather than maintaining a stationary state, the barrier between standing and motion is lowered, enabling a spontaneous, dynamic reaction to destabilization. That is, it allows a high degree of manoeuvrability or variability.

This research discusses whether such a transition is actually used in human standing by two methods: (i) mathematical analysis of the posture control model and (ii) evaluation of human control of standing sway. In the mathematical analysis, an analytical solution of the nonlinear posture control model with noise is found, and integral control and biological noise are shown to contribute to smooth transition. In the human experiment, the control state of standing motion on floors of different stability is quantitatively evaluated by using the system model. By comparing the enlargement of sway in lower stability conditions with the analytical solution of the model, transition in human standing is elucidated.

## 2. Results

### 2.1 Model analysis of sway generation mechanism

**Figure 1.**
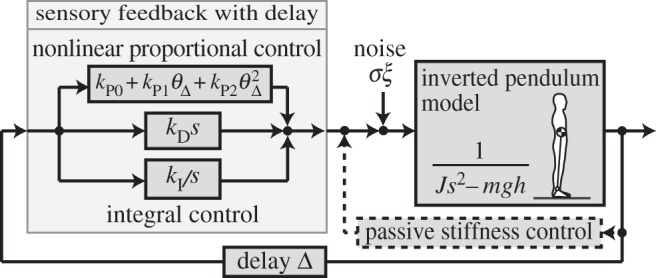
Block diagram of the control system. Feedback control comprises delayed sensory feedback and stiffness control; the stiffness control is considered as part of the sensory feedback control.

Body sway in human standing on a stable floor has been successfully explained by the intermittent control model [6–9], and simulation of a nonlinear posture control model predicted a bifurcation structure [11]. By investigating the analytical solution of the model, important factors for generating smooth enlargement of sway are discussed. The system model discussed here, proportional–integral–derivative (PID) control with third-order nonlinearity, is shown in figure 1. This model has a control structure similar to that in the event-driven intermittent control model and a mathematical structure similar to that in other research [11]. The relation between the present model and the event-driven intermittent control model is discussed in §3. The behaviour of the model is determined by the relation between linear proportional gain *k*_P0_ and body mass *m*, acceleration due to gravity *g*, length from ankle to the centre of mass (COM) *h*, inertial moment around an ankle joint *J*, derivative gain *k*_D_, and integral control gain *k*_I_. Mathematical analysis is performed for (i) the effect of integral control in the destabilization process in a linearized equation without noise, (ii) bifurcation structure in a nonlinear equation without noise, and (iii) the effect of noise on smooth transition in a nonlinear equation with noise. Details about the background of the model and the calculation process are described in the Material and methods section.

**Figure 2.**
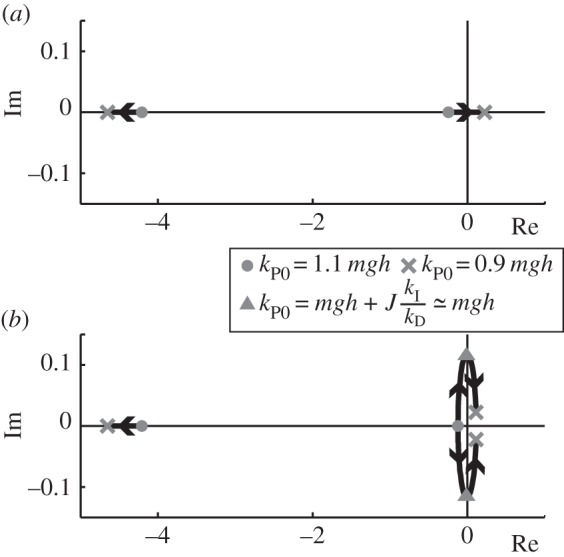
Root locus of the linearized system model without noise. (*a*) PD control model and (*b*) PID control model. Arrows show the transition of the value of poles with variable *k*_P0_ from 1.1 *mgh* to 0.9 *mgh*.

To consider the destabilization process, root locus analysis of the linearized system (proportional control gain *k*_P_=*k*_P0_) was first conducted by excluding the effect of biological noise. Figure 2*a* shows the root locus of a proportional–derivative (PD) control model, and figure 2*b* shows that of a PID control model. The parameters used to produce figure 2 reflect the identified human parameters described below (table 1). As shown in the figure, only the PID controller (figure 2*b*) has conjugate roots when the value of *k*_P0_ is around that of *mgh*, and these conjugate roots cross the imaginary axis at *k*_P0_=*mgh*+*J*(*k*_I_/*k*_D_). Because *mgh*≅593 and *J*(*k*_I_/*k*_D_)≅0.80, the destabilization point occurs at *k*_P0_=*mgh*+*J*(*k*_I_/*k*_D_)≅*mgh*. The frequency corresponding to these conjugate roots at the crossing state is $(1/2\pi )\sqrt{k_{I}/k_{D}}$. Therefore, the system has motion with this frequency when integral control is present. If the motion generated by these conjugate roots, maintained with nonlinear control, can lower the destabilization point, then cyclic motion will smoothly appear as a motion with the same frequency.

**Table 1.** Estimated parameters of the system. Values are means (s.d.) for all participants and trials.

	*k*_P0_/*mgh*	*k*_P1_/*mgh*	*k*_P2_/*mgh*
fixed floor	1.06 (0.04)	1.42 (0.34)	113.4 (45.4)
rotational floor	0.97 (0.07)	1.33 (0.40)	127.0 (43.6)

	*k*_D_/*mgh*	*k*_I_/*mgh*	*σ*
fixed floor	0.41 (0.13)	0.006 (0.001)	0.34 (0.07)
rotational floor	0.30 (0.12)	0.009 (0.006)	0.30 (0.10)

**Figure 3.**
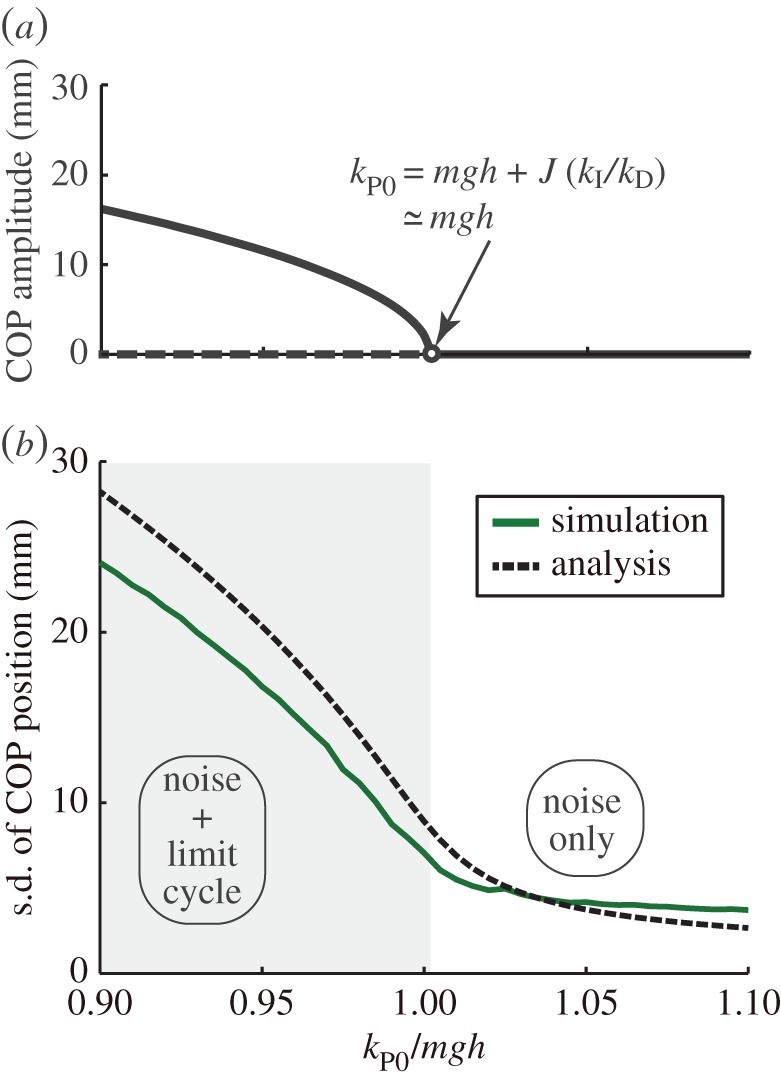
Characteristics of the system model. (*a*) Bifurcation structure of the system without noise. The solid line represents the stable solution, the dotted line represents the unstable solution and the white circle shows the bifurcation point. (*b*) Standard deviation of the COP in the system with noise. The system shows the limit cycle in the grey area. Transition between the stable stationary state and the limit cycle occurs continuously both in simulation (green line) and analytically (black dotted line).

Taking the effect of the nonlinear control into consideration, we next consider the cyclic solution of the nonlinear system (*k*_P_=*k*_P0_+*k*_P1_*θ*+*k*_P2_*θ*^2^), which excludes biological noise. Here, *θ* is the elevation angle of the body, and *k*_P1_ and *k*_P2_ are nonlinear control gains. As shown in figure 3*a*, this nonlinear system exhibits Hopf bifurcation determined by *k*_P0_, meaning that the system converges to a stable stationary state when *k*_P0_>*mgh*+*J*(*k*_I_/*k*_D_)≅*mgh*, and this fixed solution is destabilized and a cyclic solution is generated when *k*_P0_<*mgh*+*J*(*k*_I_/*k*_D_)≅*mgh*. The frequency of the cyclic solution is $(1/2\pi ){\sqrt{k}}_{I}/k_{D}$. This result indicates that if *k*_P0_ for human control is maintained around the bifurcation point *mgh*, a large difference in the amplitude of body sway before and after the bifurcation is potentially observed. Moreover, if the variation in *k*_P0_ is small, then the frequency $(1/2\pi )\sqrt{k_{I}/k_{D}}$ exists both before and after bifurcation. This observation of bifurcation is subsequently discussed through identification of the system model.

The bifurcation structure shows the enlargement of sway according to stability. However, on another front, figure 3*a* shows rapid change in the sway amplitude at the bifurcation point, which is difficult to believe as a human behaviour. We considered that ignoring biological noise might cause this rapid change, so we performed mathematical analysis including the effect of noise. As a result of analysing the equation with noise, we found that the variance of COM 〈*θ*^2^〉 changes according to the equation

$$\langle \theta^{2}\rangle =\frac{1}{2}k_{P2}\left\{-k_{P0}+\text{mgh}+J\left(\frac{k_{I}}{k_{D}}\right)+\sqrt{\left(-k_{P0}+\text{mgh}+J{\left(\frac{k_{I}}{k_{D}}\right)}^{2}\right)+4k_{P2}\sigma^{2}}\right\}$$

(see Material and methods for the effect of biological noise). Figure 3*b* shows the result of analysis including approximately the same magnitude of human noise [4]. In this figure, the green line represents the simulation results and the black line represents the analysis results. The figure shows that the amplitude of body sway continuously varies around *k*_P0_≅*mgh* in both simulation and analysis. Therefore, body fluctuation generated by biological noise absorbs the rapid transition from a stationary state to cyclic motion.

From the above, the analysed model has the following characteristics. First, in accordance with the decrease in the linear proportional gain *k*_P0_, the stationary state destabilizes and cyclic motion appears. Second, the transition from a stable state through a decrease in *k*_P0_ does not show a rapid change of motion; it changes smoothly, owing to the effect of biological noise. In the following subsection, the existence of such a smooth bifurcation mechanism in human posture control is discussed. The information used for this is obtained by measuring human standing under different stability conditions and by quantitatively comparing the control state of measured human sway and the analysed system model.

### 2.2 Standing motion on fixed and rotational floors

**Figure 4.**
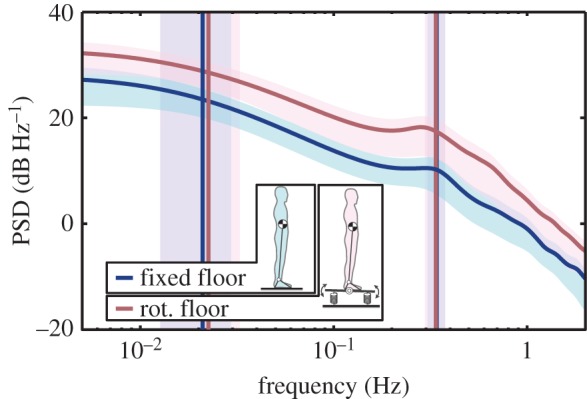
Power spectrum density (PSD) of the centre of pressure (COP). Curved lines are the PSD of measured COP derived by the maximum entropy method. Blue and red vertical lines indicate the characteristic frequencies of the PSD, calculated by the minimum frequency of the second differential values. Blue and red areas are the standard deviations of the PSD and the characteristic frequencies.

Standing motion was measured on floors with different stabilities, that is, fixed and rotational floors, and sagittal swaying motion was investigated. First, sway amplitude, defined as the range of centre of pressure (COP), was calculated. The average (s.d.) of COP motion was ±22.6 (7.2) mm on the fixed floor and ±41.0 (12.4) mm on a rotational floor. Swaying motion was thus approximately twice as large on a rotational floor as on a fixed floor. Next, the power spectrum density (PSD) of the swaying motion was calculated by using the maximum entropy method (MEM) [12,13], as shown in figure 4. MEM was used in this research because it is unlikely to be unstable when there are few data with respect to the frequency, that is, when calculating the PSD for low-frequency ranges (see electronic supplementary material, figure S1, for a comparison of PSD calculated by fast Fourier transform and MEM).

Upon searching within the characteristic frequencies of the obtained PSD, clear peaks were found at 0.34 (0.04) Hz for both fixed and rotational floor conditions as the average (s.d.) of data from all subjects. The results of two-way ANOVA for floor conditions and participants did not show significant differences in peak frequencies according to the floor condition (*p*=0.43), and significant differences between subjects were not found (*p*=0.25). Moreover, another frequency component, indicated by changes in the slope of the PSD, was found, with a frequency of about 0.02 Hz for every subject (see Material and methods for details on searching for frequency components). The frequency of this component was 0.02 (0.01) Hz for both fixed and rotational floor conditions, taken as the average (s.d.) of data from all subjects (see electronic supplementary material, figure S2, for the results of characteristic frequencies of all data). These frequencies were not dependent on the floor condition (*p*=0.70), but significant differences between subjects were found (*p*<0.01) by two-way ANOVA. However, because this discovered low-frequency component does not yield clear peaks and the frequencies differ significantly between subjects, the existence of such a low-frequency component cannot be confidently asserted. Despite this, the result that a characteristic frequency of less than 0.1 Hz is found at only a single frequency for each subject supports the possibility that this frequency component actually exists. Technical difficulties of measuring standing for a long duration are known (see Discussion section for further discussion about the peaks of frequencies), and these technical issues may be the reason that finding clear peaks is difficult.

Broadly, swaying motions for different floor stabilities showed similar frequency distributions and a different range of motion. Using these characteristics, we compared sway amplitude and control parameters to verify the underlying mechanism of enlargement of sway.

### 2.3 Bifurcation in human standing

The analysed control mechanism predicts an increase of sway amplitude owing to the decreasing linear stability gain *k*_P0_, from bifurcation. By decreasing the stability of the standing floor, *k*_P0_ can be equivalently reduced and, if the analysed mechanism is consistent with human control, then the transition may be observed in human experiments when *k*_P0_ falls below *mgh*. To test this hypothesis, control gains of motion were identified for floors of different stability: a fixed floor and a rotational floor. Then, the value of the identified *k*_P0_ was compared with the amplitude of sway represented by the standard deviation of COP.

**Figure 5.**
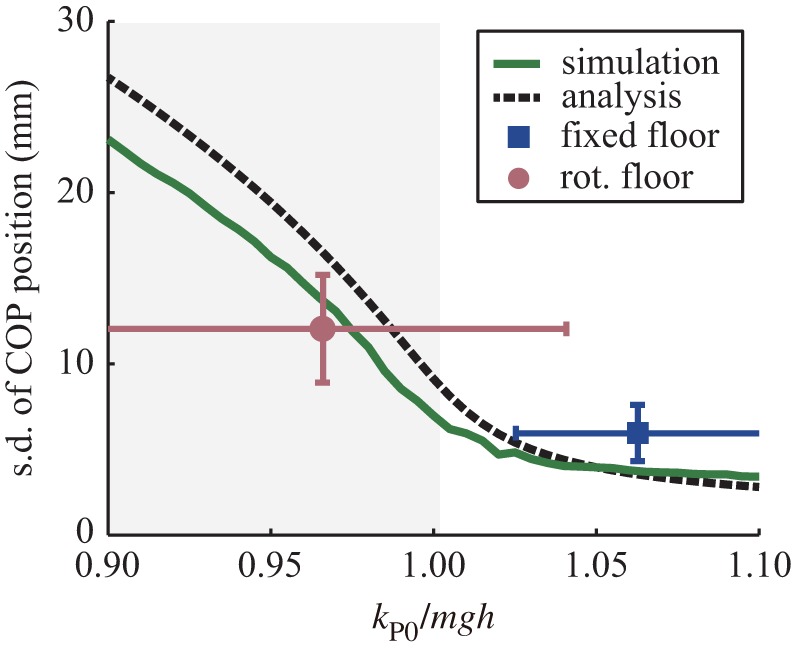
Comparison of the characteristics of control gain and sway amplitude (s.d. of COP) of the model and experimental results. Blue and red points are the means of the results for fixed and rotational floors, respectively. Lines around the points show the s.d. of the gain and sway amplitude.

Control parameters were searched by comparing PSD of the experiment with that of the model simulation (see Material and methods for identification of the model parameters; the fitness of the model simulation is shown in electronic supplementary material, figure S3). The identified parameters are shown in table 1. The identified values of *k*_D_ and *k*_I_ were consistent with those suggested by high-gain PD control [14,15] and PID control [16], and the magnitude of the noise *σ* was in the range of the estimated biological noise [4]. The identified values of *k*_P0_ were 1.06 *mgh* for a fixed floor and 0.97 *mgh* for a rotational floor. This means that the standing state is maintained under both conditions around the bifurcation point, *k*_P0_≅*mgh*, which is the stability limit of linear control. Moreover, on average the value of *k*_P0_ on the rotational floor was lower than that on the fixed floor, and lower than *mgh* on a rotational floor. The calculated frequency around the bifurcation point $(1/2\pi )\sqrt{k_{I}/k_{D}}$ was 0.02 (0.00) Hz under the fixed floor conditions and 0.03 (0.01) Hz under the rotational floor conditions. Both frequencies were almost equal to those observed experimentally. Based on the identified parameters, the relation of the magnitude of body sway and control gain *k*_P0_ was plotted and compared with the analytical results, as shown in figure 5. In this figure, the standing states for the fixed floor and rotational floor conditions were separated into a stationary state (the white area) and a cyclic motion state (the grey area). The increased body sway accompanied by decreasing gain, *k*_P0_, was confirmed to occur as indicated in the analytical solution of the system model.

## 3. Discussion

We examined the bifurcation-induced transition of human sway depending on floor stability, and investigated a human posture control mechanism by performing the following analyses: analysis of the bifurcation structure and the contribution of biological noise, measurement of human sway on different floor conditions, and quantitative evaluation of the human control gains and bifurcation. The following characteristics were found. (i) The human standing control model (third-order nonlinear control model with PID controller) exhibits bifurcation from a stationary state to cyclic motion when the value of *k*_P0_ falls below *mgh*+*J*(*k*_I_/*k*_D_), which is approximately *mgh*, and the transition through the bifurcation is smoothly achieved by the effect of biological noise. (ii) Human body sway includes a low-frequency component with frequency of approximately 0.02 Hz on both fixed and rotational floors, and the sway amplitude is approximately twice as large under the rotational floor condition. (iii) Linear control gain *k*_P0_ is maintained at approximately *mgh* for both fixed and rotational floors; it is higher than *mgh* on fixed floors and lower than *mgh* on rotational floors. The increase in sway caused by decreasing *k*_P0_ quantitatively matched the analytically obtained bifurcation. Moreover, the frequency predicted by the model was similar to the experimentally indicated frequency of 0.02 Hz. From these results, we consider that humans generate a large body sway through floor instability in accordance with the bifurcation.

The analysed mechanism shows that humans actively use the saddle structure of body dynamics in composing a limit cycle. The utilization of the saddle structure of body dynamics in quiet standing has also been pointed out for the intermittent control model [7,17]. The utilization of body dynamics is effective in allowing control to be sensitive to the environment and, as indicated by the present research, it allows spontaneous transition from a stable state to a moving (limit cycle) state depending on standing conditions. Such a dynamic response to the environment, instead of maintaining a stationary state, shows a human strategy of posture control that gives high priority to manoeuvrability.

### 3.1 Relation between the nonlinear model and the intermittent control model

This research used a continuous nonlinear control model with third-order nonlinearity. We used a continuous model for control because it has advantages in the mathematical analysis for clarifying the mechanism of nonlinear posture control and parameter identification based on experimental data. Since the third-order nonlinearity can be considered to be a continuous approximation to discrete control, our approach does not rely on making a distinction between continuous or discrete control. Nonlinear control in the present research has a mechanism similar to that of on–off (event-driven) intermittent control models [8] for generating large sway; our model approximates the nonlinearity of event-driven intermittent control due to discrete structure by nonlinearity due to the third-order nonlinear function. The following describes this relation between the event-driven intermittent control model and the nonlinear control model we used.

**Figure 6.**
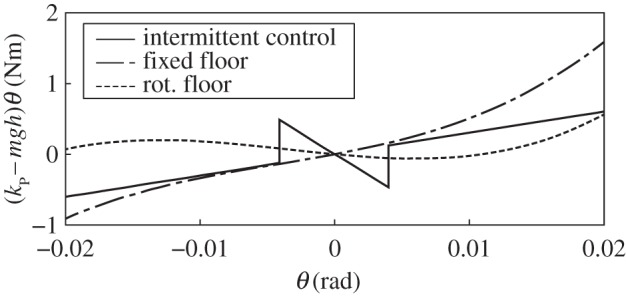
Comparison of the effect of control between event-driven intermittent control and nonlinear control of the present research. The vertical axis of the figure shows the deviation between the moment working for falling down and the control torque by intermittent control (solid line) and nonlinear control of the present research (dash-dotted line and dotted line). Parameters for the intermittent control are those from Asai *et al*. [8] and parameters for the dash-dotted line and dotted line are those identified from the motion on fixed and rotational floors, respectively.

The main reason for introducing the intermittent control for the human standing model is because large body sway of approximately 20 mm cannot be explained by continuous PID control with haemodynamic noise [5]. To explain this large sway, event-driven intermittent control has a dead zone in the standing state where active feedback control does not work, and sway by noise is only weakly sustained by passive stiffness control. Let us briefly consider the function of this dead zone for generating large sway from the viewpoint of stability. The stability condition of standing control is that when it is considered as linear control (a piecewise, linearized system around the dead zone), *k*_P_>*mgh* as in the Results section (the integral control gain *k*_I_ is supposed to be sufficiently small). System stability is thus basically determined by the magnitude relation between the proportional control torque *k*_P_*θ* and body moment for falling *mghθ*. When the deviation (*k*_P_−*mgh*)*θ* between torque and moment is graphed (figure 6), the system becomes stable if the slope of (*k*_P_−*mgh*)*θ* over *θ* is positive and unstable if negative. The solid line in the figure is (on–off type) intermittent control with passive control gain 0.8 *mgh*, active (intermittent) control gain 0.25 *mgh*, and a dead zone range 0.004 rad (these parameters were used in [8]). For simplicity, the dead zone at the centre of the *θ*–$\dot{\theta }$ phase plane is considered, and any dead zone depending on the falling direction and velocity is not considered. Generation of large sway under this condition can be explained by weak restriction of sway due to a small or negative slope with small *θ*, and stability due to positive slope with large *θ* (sway generation due to a limit cycle will simultaneously occur if a negative slope exists).

The nonlinear model used in the present research has a similar structure using a third-order nonlinear function. The dash-dotted line and dotted line in figure 6, respectively, show the deviation (*k*_P_−*mgh*)*θ* when using the parameters identified by motion on fixed and rotational floors. A discrete function can be considered (by Taylor decomposition) as a superposition of control with high-dimensional nonlinearity, so our function can be considered as a third-order approximation of the discrete model. The structure of the intermittent control model of small or negative slope at small *θ* and positive slope at large *θ* is maintained in the third-order nonlinear control as in the figure and, therefore, both controls are considered to have similar control characteristics for standing control. Conversely, mechanisms discovered in the present research, such as the enlargement of sway due to bifurcation, are potentially maintained in the intermittent control model. The approach here is thus considered applicable to systems implementing the event-driven intermittent control model.

### 3.2 Comparison of identification results with those from conventional models

The control gain of a standing person has been identified by Peterka [16,18]. Peterka numerically evaluated the control gain of a PID control from the frequency response in order to estimate the gains. By comparing these values with the values obtained in the present research (table 1), we see that the derivative control gain *k*_D_=0.4–0.45 *mgh* obtained by Peterka is similar to our value, *k*_D_=0.30–0.41 *mgh*. For the integral gain, the result of *k*_I_=0.02 *mgh* [16] is not far from our result, *k*_I_=0.006–0.009 *mgh*. The obtained value of *k*_P0_, separated from *k*_P_ by the nonlinear members *k*_P1_ and *k*_P2_, is smaller than *k*_P_ (*k*_P0_≈1.0 *mgh*), and the difference supports the presence of nonlinear controls. Thus, the numerical results obtained in the present research are considered reasonable in comparison with those from prior research.

In the high-gain PD control model [14,15], the condition of derivative control gain needed to realize continued electromyography (EMG) readings has been estimated. In the condition proposed by Masani *et al*. [14,15], the ratio of proportional control to derivative control, *k*_P_/*k*_D_, should be in the range 1.0–5.57. In this condition, if *k*_P_ is 1.0 *mgh*, then *k*_D_ becomes 0.18–1.0 *mgh*. This condition is satisfied by the obtained results (table 1), which have *k*_D_=0.30–0.41 *mgh*. Therefore, the proposed model satisfies the conditions of high-gain PD control, and so our model also explains the phenomenon of continuing EMG readings.

Bottaro *et al*. [5] considered body sway from the viewpoint of the magnitude of noise and showed the necessity of nonlinear control in human standing. They estimated that at least 5 Nm of ankle torque from biological noise would be required to generate the same amplitude of body sway as given by a PID control model. This amplitude is more than 10-fold the level of haemodynamic noise, which has been estimated to be about 0.4 Nm around the ankle joint [4]. The obtained magnitude of noise in the present research (table 1) is *σ*=0.30–0.34 Nm, so the proposed model has the ability to produce large sway with small noise, similar to an event-driven intermittent control model [7].

### 3.3 Effect of delay and noise

An inevitable problem for human posture control is delay and noise. In human standing control, the effect of delay is suppressed by generating slow cyclic movement. Moreover, the results here indicate that the effect of noise is, in contrast, used for control in the sense that rapid change of the sway amplitude by Hopf bifurcation is smoothed by the small noise-induced sway at the stable stationary state and by the increase of the noise-induced sway around the bifurcation point through the decreased control gain, *k*_P0_. The use of noise for human standing was predicted by Eurich & Milton [6], such that transition between different stable states occurs depending on the noise level. This noise is known to be used for improving sensitivity in the human brain [19], neurons [20] and other animals [21] by a mechanism known as stochastic resonance [22]. In the present research, another method for using noise is considered: to smooth the transition of the standing state. This function of noise has been discussed in some prior research. For example, the threshold may be smoothed by the effect of dithering [23], and nonlinear activity by spiking neurons can be linearized by the effect of noise [24]. Such a mechanism is known to occur in a biological system, with the dynamics of the pupil light reflex clamped via piecewise constant feedback [25]. Here, the pupil area is changed smoothly despite discontinuity in the discrete feedback. This is considered to be due to random variations, namely, noise. These research results support the idea that the use of noise for smoothing, which is considered to be its function in posture control in the present research, is possible in a biological system.

### 3.4 Existence of a limit cycle

As a frequency characteristic of sagittal human sway, a motion with approximately 0.4 Hz has been discussed in several studies [26–29]. This motion is observed to be ballistic, and it can reflect the no-control state [30,31]. As a slower motion, oscillatory motion with a frequency lower than 0.1 Hz has been reported [32,33], but the origins of this slower motion have not been comprehensively discussed. An event-driven intermittent control model predicts the existence of ballistic motion by including a no-control state and the existence of limit cycles for larger and slower behaviour. Thus, if we adopt this viewpoint, the slower (less than 0.1 Hz) motion will correspond to the limit cycle. In the present research, the discussed system model showed a limit cycle with peak frequency of 0.02 Hz, and measured motion showed a peak frequency at 0.34 Hz and indicated the possible existence of a low-frequency component of approximately 0.02 Hz.

Although our results show that the slope of the PSD is changing at around 0.02 Hz, a clear peak is not seen at that frequency. One possible reason for this is that technical difficulties arise in analysing low-frequency motion of standing. In particular, to analyse a low-frequency component, quiet standing has to be measured for a long duration. Such experiments to investigate long-duration standing have been attempted in several studies [34–36]. To measure the standing motion in a natural state, the standing environment has included various conditions, such as continuous questioning during standing [37] and allowing conversation [35]. Despite such attempts, mechanisms within the low-frequency range are not well understood. By taking this difficulty into consideration, the results of the present research, in which low-frequency components do not show clear peaks, are not unusual. Instead, the obtained change in the slope supports the existence of a characteristic component around this frequency, even though this cannot be asserted with great confidence. Moreover, the result that the frequency was similar between the system model and the experiment also supports the underlying frequency component and its mechanism.

### 3.5 Integral control

The present system model included integral control, in contrast with the conventional nonlinear standing model [8,11]. The integral control resulted in three roots, and conjugate roots appeared. These conjugate roots (figure 2*a*) generated an eigenfrequency around the transition point, and were connected to the limit cycle state through the Hopf bifurcation. This mechanism is reflected in the observed human behaviour as a transition without frequency change. The existence of an integral function in neural control has been discussed, centring on the motion control of saccades [38,39] and the integral function is present in the circuit of parallel-fibre Purkinje cells in the cerebellum [40]. These results indicate that such an integral function may be used for the transition of the standing state.

### 3.6 Possible application for the mechanism of ataxic behaviour

Neurological diseases can change the properties of body sway, and the analysed mechanism may be related to this change. For example, the amplitude of body sway in individuals with Parkinson’s disease is known to be small [1,2]. From figure 5, a decrease in sway represents an increase in the control gain (*k*_P0_), indicating that the control in Parkinson’s disease weighs heavily on stability. This also means the barrier to reach the bifurcation point or the initiation of movement is increased. Such an increase in difficulty for initializing movement potentially reflects the freezing of gait observed in Parkinson’s disease. The present research succeeds in quantitative evaluation of sway from a control model, and thus possibly allows quantitative evaluation of symptoms of diseases related to disorders in neural control.

## 4. Material and methods

### 4.1 Model

In this research, rotational motion of the COM around the ankle joints is modelled as an inverted pendulum on the sagittal plane:

4.1 $$J\ddot{\theta }=\text{mgh}\theta +\tau +\sigma \xi ,$$

where *θ* is the elevation angle of the pendulum, *m* is body mass, *g* is acceleration due to gravity, *h* is the length from ankle to the COM and *J* is the inertial moment around an ankle joint. Control torque *τ* is given at the ankle, and biological noise *σξ* is modelled as Gaussian white noise, with *ξ* a normalized noise and *σ* its magnitude.

Posture control is composed of stiffness control [41] and sensory feedback (figure 1). Stiffness control is due to the spring-like nature of muscles (and collateral effects from the stretch reflex), and sensory feedback control is due to cognitive response to sensations. Stiffness control functions in real time and the sensory feedback is obtained with a lag of about 150 ms for cognition. Here, gains in stiffness and sensory feedback control were experimentally identified from response frequencies [18], in which sensory feedback had a gain 10-fold greater than that of stiffness control. Moreover, we focused on the 0.02 Hz motion frequency, where a cycle is 50 s, and a delay of 150 ms is comparatively small. Based on these notions, stiffness control is approximated to be part of sensory feedback control in this research.

As a sensory feedback control, the PID control model

4.2 $$\tau =-k_{P}\theta_{\Delta }-k_{D}{\dot{\theta }}_{\Delta }-k_{I}\int \theta_{\Delta }dt$$

has been proposed [18,42]. This model replicates many features of human standing motion, including the stabilogram diffusion function [16,43,44]. In equation (4.2), *k*_P_, *k*_D_, and *k*_I_ are the proportional, derivative and integral control gains, respectively, and *θ*_Δ_ represents angle *θ* from the COM with a delay. As shown in the result of linearized analysis of the system, the root locus of PID control has conjugate roots (figure 2*b*), whereas that of PD control does not (figure 2*a*). The instability of these conjugate roots contributes to the bifurcation, thus PID control is used in this research.

Control parameters of a linear PID model (equation (4.2) with constant *k*_P_) have been quantitatively discussed [16,18], and Bottaro *et al*. [5] pointed out that the minimum noise necessary to generate a typical body sway amplitude of 0.181^°^ by the linear PID model was 5 Nm. This value is 10-fold larger than the haemodynamic noise generated by heartbeats (0.4 Nm [4]), and haemodynamic noise is the largest biological noise during standing. Therefore, to explain the amplitude of body sway, the control system requires some nonlinearity. This problem of large sway is solved in some other models, such as the event-driven intermittent control model [7,17,18,25,45] and the throw-and-catch control model [30,33,46–48]; these models contain an uncontrolled state around the vertical state (centre of the COP distribution), in which unreduced noise generates a large sway. A bifurcation structure was found in a simulation study of a continuous nonlinear control model [11]. For clarity in the analysis and parameter identification, a continuous control model that contains nonlinear structure based on the event-driven intermittent control model is adopted in the present research. The mathematical structure of our model is almost equivalent to that used in the previous study [11], and the relation between our model and the event-driven intermittent control model is discussed in Discussion section. In this model, the proportional control *k*_P_ is represented by a third-order function of *θ*:

4.3 $$k_{P}=k_{P0}+k_{P1}\theta_{\Delta }+k_{P2}\theta_{\Delta }^{2}.$$

We show analytically that this system model possesses Hopf bifurcation induced by *k*_P0_ and generates large body sway as a cyclic motion.

### 4.2 Model analysis

The mechanism of sway generation in the model is analysed by three steps: analysis of the destabilization process by using a linear system without noise (*k*_P_=*k*_P0_,*σ*=0); analysis of cyclic solution using a nonlinear system without noise (*σ*=0); and analysis of the noise effect.

#### 4.2.1 Fundamental equation

The model equations (equations (4.1)–(4.3)) can be written together as

4.4 $$J\ddot{\theta }=\text{mgh}\theta -(k_{P0}+k_{P1}\theta_{\Delta }+k_{P2}\theta_{\Delta }^{2})\theta_{\Delta }-k_{D}{\dot{\theta }}_{D}-k_{I}\int \theta_{\Delta }dt+\sigma \xi .$$

The objective motion of the analysis is sufficiently slow, less than 1 Hz, compared with the sensory feedback delay of less than 200 ms [7,18,49]. Thus, the delay is neglected in the analysis (*θ*_Δ_=*θ*). Moreover, the second-order nonlinear factor, *k*_P1_, contributes only to the deviation from the COM, so it can be neglected for the sake of simplicity (*k*_P1_=0). Thus, the equation becomes

4.5 $$J\ddot{\theta }≅\text{mgh}\theta -(k_{P0}+k_{P2}\theta^{2})\theta -k_{D}\dot{\theta }-k_{I}\int \theta dt+\sigma \xi$$

and by setting $\psi =\dot{\theta }$ and η=∫θdt, the equation can be written as

4.6 $$J\dot{\psi }≅\{(\text{mgh}-k_{P0})-k_{P2}\theta^{2}\}\theta -k_{D}\psi -k_{I}\eta +\sigma \xi .$$

Analysis is performed using equations (4.5) and (4.6).

#### 4.2.2 Analysis of destabilization process

By simulating the model (equation (4.5)) with various linear proportional gains, *k*_P0_, two steady states were found: a stationary state when *k*_P0_>*mgh* and a limit cycle when *k*_P0_<*mgh*. To understand the characteristics of the solution around the transition point at *k*_P0_≅*mgh*, first the linear response around *k*_P0_≅*mgh* of equation (4.5) with *k*_P2_=0 was investigated. When *k*_P0_ was set to be 1.1 *mgh* and gradually reduced to 0.9 *mgh*, the poles of the transfer equation moved along the arrows shown in figure 2. In the case of PID model (figure 2*b*), one pole was located on the real axis with a strictly negative value (the stable pole), and the other two were complex conjugates of each other. The conjugate poles had a negative real value when *k*_P0_=1.1 *mgh*, although they crossed the imaginary axis at *k*_P0_≅*mgh* and became unstable. The crossing point of the imaginary axis can be obtained from the frequency transfer function ( *jω*)

4.7 $$G(\;j\omega )=-k_{D}\omega^{2}+k_{I}-j\omega (\text{mgh}-k_{P}+J\omega^{2})=0.$$

At that point, *k*_P_=*mgh*+*J*(*k*_I_/*k*_D_) and the angular frequency $\omega =\sqrt{k_{I}/k_{D}}$. Thus, the limit cycle can be generated as the effect of these conjugate poles, whose instability at *k*_P0_<*mgh*+*J*(*k*_I_/*k*_D_) is sustained by the effect of the nonlinear control, *k*_P2_.

#### 4.2.3 Analysis of cyclic solution

To investigate the convergence around *k*_P0_≅*mgh*, including the effect of the nonlinear control, *k*_P2_, the conditions necessary for a cyclic steady state and for stability are discussed. To focus on the solution by conjugate poles in equation (4.5), the following general form of the solution is considered:

4.8 $$\left\{ \begin{array}{l} \text{solution:}\;\theta =\text{a}e^{j\omega t}+a^{*}e^{-j\omega t}\\ \dot{a}e^{j\omega t}+{\dot{a}}^{*}e^{j\omega t}=0. \end{array}\right.$$

Here, for the purpose of simplicity, the integral term is approximated by

4.9 $$\int \theta \text{d}t=\frac{1}{j\omega }(a{\text{e}}^{j\omega t}-a^{*}{\text{e}}^{-j\omega t}).$$

Substituting equations (4.8) and (4.9) into equation (4.5) and integrating both members for one cycle, the equation becomes

4.10 $$\dot{a}=-\frac{1}{2J\omega }\left(k_{D}\omega -\frac{k_{I}}{\omega }\right)a-\frac{j}{2J\omega }(J\omega^{2}+\text{mgh}-k_{P0}-3k_{P2}{\text{aa}}^{*})a$$

(see appendix A for detailed calculation process). Substituting *a*=*re*^*jϕ*^, a cyclic solution is extracted. This operation is equivalent to the comparison of the real and imaginary part of equation (4.10) with $\dot{a}=\dot{r}e^{j\phi }+j\dot{\phi }re^{j\phi }$, so that the following relationship can be obtained:

4.11 $$\left\{ \begin{array}{l} \dot{r}=-\frac{1}{2J\omega }\left(k_{D}\omega -\frac{k_{I}}{\omega }\right)r\\ \dot{\phi }=-\frac{1}{2j\omega }(J\omega^{2}+\text{mgh}-k_{P0}-3k_{P2}r^{2}). \end{array}\right.$$

From the above equations with $\dot{r}=0,\dot{\phi }=0$ a steady amplitude for the cyclic solution can be obtained:

4.12 $$r=\left\{ \begin{array}{l} 0\\ \sqrt{\left(1/3k_{P2})(-k_{P0}+\text{mgh}+J(k_{I}/k_{D})\right)} \end{array}\right.$$

This equation shows that the fixed solution *r*=0 (i.e. *θ*=0) always exists, and a cyclic solution simultaneously exists when *k*_P0_<*mgh*+*J*(*k*_I_/*k*_D_). Moreover, angular frequency *ω* of the cyclic solution is obtained as $\omega =\sqrt{k_{I}/k_{D}}$.

To consider the stability of the fixed solution *θ*=0, equation (4.6) is linearized around *θ*=0. Then the equation becomes

4.13 $$\left[\begin{array}{c} \dot{\eta }\\ \dot{\theta }\\ \dot{\psi } \end{array}\right]=\left[\begin{array}{ccc} 0 & 1 & 0\\ 0 & 0 & 1\\ -\frac{k_{I}}{J} & \frac{\text{mgh}-k_{P0}}{J} & -\frac{k_{D}}{J} \end{array}\right]\left[\begin{array}{c} \eta \\ \theta \\ \psi \end{array}\right],$$

and the characteristic equation becomes

4.14 $$\lambda^{3}+\frac{k_{D}}{J}\lambda^{2}-\frac{1}{J}(\text{mgh}-k_{P0})\lambda +\frac{k_{I}}{J}=0.$$

From this equation, the stability condition of the solution, *θ*=0, becomes

4.15 $$k_{P0}>\text{mgh}+J\frac{k_{I}}{k_{D}}.$$

#### 4.2.4 Effect of biological noise

Next, the features of a system that includes biological noise (i.e. *σ*≠0) are investigated and the effect of the noise is discussed.

To analyse the transition of COM with variance 〈*θ*^2^〉, we observe that *θ*^2^ and *θ*^3^ converge more quickly than *θ* does, so the nonlinear term of *k*_P_ is linearized by using the average of *θ* (mean field approximation [50]). This yields

4.16 $$k_{P}≅k_{P0}+k_{P2}\langle \theta^{2}\rangle ,$$

which expresses the average and 〈*θ*^2^〉 becomes the variance because 〈*θ*〉≅0. Then, equation (4.6) can be described by the Langevin equation

4.17 dX=AX dt+dW,

where

$$X=\left[\begin{array}{c} \eta \\ \theta \\ \psi \end{array}\right],\;A=\left[\begin{array}{ccc} 0 & 1 & 0\\ 0 & 0 & 1\\ -\frac{k_{I}}{J} & \frac{mgh-k_{P0}}{J} & -\frac{k_{D}}{J} \end{array}\right],\;\text{d}W=\left[\begin{array}{c} 0\\ 0\\ \frac{\sigma }{J}\xi \end{array}\right].$$

At this time, the correlation matrix about X and the magnitude of noise, *D*, are given by

4.18 $$R=\langle XX^{T}\rangle =\left[\begin{array}{ccc} \left\langle \eta^{2}\right\rangle & \left\langle \eta \theta \right\rangle & \left\langle \eta \psi \right\rangle \\ \left\langle \eta \theta \right\rangle & \left\langle \theta^{2}\right\rangle & \left\langle \theta \psi \right\rangle \\ \left\langle \eta \psi \right\rangle & \left\langle \theta \psi \right\rangle & \left\langle \psi^{2}\right\rangle \end{array}\right],\;D=\langle \text{d}W\;\text{d}W^{T}\rangle =\left[\begin{array}{ccc} 0 & & \\ & 0 & \\ & & {\left(\frac{\sigma }{J}\right)}^{2} \end{array}\right],$$

respectively, which have the following relationship by the fluctuation-dissipation theorem:

4.19 $$AR+RA^{T}=-2D.$$

Then, the variance of COM 〈*θ*^2^〉 becomes

4.20 $$\langle \theta^{2}\rangle =\frac{1}{2k_{P2}}\left\{-k_{P0}+\text{mgh}+J\left(\frac{k_{I}}{k_{D}}\right)+\sqrt{\left(-k_{P0}+\text{mgh}+J{\left(\frac{k_{I}}{k_{D}}\right)}^{2}\right)+4k_{P2}\sigma^{2}}\right\}$$

(see appendix B for detailed calculation process). This equation provides an analytical solution for the variance. Then the standard deviation of COP is calculated from the analytical result (equation (4.20)) and the simulation with the control gain, *k*_P0_, for discussing the bifurcation structure of the system.

The accuracy of the analytical solution is verified by comparison with dynamical simulation. The parameters used for the dynamical simulation are based on a human experiment described later (table 1). The parameter values for the inverted pendulum model are as follows: *m*=63.7 kg, *g*=9.8 m s^−2^, *h*=0.95 m, and *J*=57.3 kg m^2^. The parameter values for the control parameters without *k*_P0_ are the values listed for the fixed floors in table 1. Moreover, to compare the simulated angle, *θ*, from the COM with the angle from the measured value of the COP, *θ* is converted using the relationship $\text{COP}=h\theta -(h_{e}h/g)\ddot{\theta }$ [51]. Here, *h*_*e*_ is the distance from the ankle to the COM modified to consider the deviation of mass, calculated as *h*_*e*_=1.15 *h* [5]. Numerical calculation of the stochastic differential equation for the simulation is performed by using the Euler–Maruyama method.

### 4.3 Experimental method

The experiment was performed under two floor conditions: fixed and rotational floors (figure 4). In the fixed floor condition, participants stood on a force plate with their legs open to shoulder width. They were asked to hold their posture for 360 s, and the motion of the COP was measured as a time series. In the rotational floor condition, they stood on a platform that had variable stability in the sagittal plane. The platform possessed a rotational shaft and was connected to the floor by springs. The stiffness of each spring was 10 N mm^−1^. Two springs were located in front of the participant and two behind. As the effect, the torque transferred from the ankle to the floor is approximately halved (see §4.4). The participants were 8 men and 1 woman; 6 participants completed 4 trials each and 3 participants completed 3 trials each.

Measurement of COP was performed with a force plate (Tec Gihan TF-4060-A), and measurement of the tilting floor was performed with a rotary encoder (MEH-85-18000P, resolution 18 000 pulses per round). Measurement frequency was 200 Hz. The experiment was performed in a quiet room, and participants wore sound-muffling ear protection for silence. Participants were healthy persons between 21 and 25 years old, with heights from 153 to 180 cm and body masses from 42 to 76 kg. Participants closed their eyes and crossed their arms in front of their chest during measurement. The experiment was performed with the participants’ eyes closed to control for the effect of individual sensory information so as to capture the characteristics of the control system under simple conditions. The effect of standing conditions, such as arm position and height, was investigated for three participants as a preliminary experiment, and COP distribution was not significantly different between conditions (*p*=0.55, by ANOVA).

### 4.4 Determination of the spring coefficient of the rotational floor

**Figure 7.**
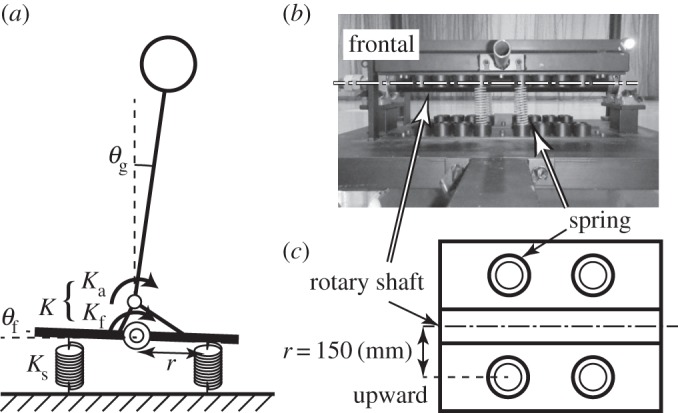
Detail of the rotational floor. (*a*) Schematic of the rotational floor on the sagittal plane and variables for the calculation of the effect of springs. (*b*) A photograph of the rotational floor from the front. The dotted line shows the position of the rotational shaft. (*c*) Schematic of the floor from above. Circles represent the positions of springs.

A rotational floor is equipped with springs having spring coefficient *K*_s_ at distance *r* either side of the rotational shaft, as shown in figure 7. When the floor tilts by a small angle, *θ*_f_, the spring length changes by $r\sin\theta_{f}≅r\theta_{f}$ and thus the springs generate torque *τ*_f_=*K*_s_*r*^2^*θ*_f_ around the rotational shaft. This corresponds to the system having springs with

4.21 $$K_{f}=K_{s}r^{2}$$

around the ankle.

For the purpose of simplification, the rotational shaft and the ankle are taken as located at the same point (i.e. the height of the foot is neglected) and the rotational reaction force of the floor is considered to be generated by the spring of *K*_f_, so the spring force around the ankle can be determined as a coupled spring force by

4.22 $$K=\frac{K_{a}K_{f}}{K_{a}+K_{f}}.$$

The spring coefficient, *K*_s_, is obtained from this relationship so that the torque around the ankle is half of this value. If *m*=60 kg, *h*=0.9 m, *r*=150 mm and *g*=9.8 m s^−2^, then *K*_s_ is calculated to be

4.23 $$K_{s}=\frac{\text{mgh}}{r^{2}}=23.5.$$

In the present research, two springs of 10 N mm^−1^ are used in parallel for constituting a 20 Nm spring, and these are installed on both sides of the rotational shaft, so a total of four springs of 10 N mm^−1^ are installed.

### 4.5 Data analysis and statistics

From the measured COP, the amplitude and power spectrum of body sway is calculated. The range of sagittal body sway, computed as the average and standard deviation of every subject and trial, is obtained as the amplitude of body sway. The power spectrum of the sway is calculated by MEM of COP. To consider the slow element of body sway, a frequency lower than 1 Hz is used as the upper limit of the frequency. Moreover, the 360 s measurement duration can provide measurements to a lower frequency limit of approximately 0.005 Hz. The calculation of MEM is performed by the Burg method [12,13] with dimensions at 512. The obtained power spectrum was searched for characteristic frequency components. Frequency components yield clear peaks when they clearly affect the motion. However, in some cases, such as when the damping factor is quite large, they will change only the slope of the PSD. Thus, the PSD was examined for places where the slope of the frequencies changes most sharply. To consider the changes in slope, a second-order differential of the PSD is calculated for the frequency domain, and those frequencies with a minimum peak (i.e. a maximum change in the negative direction) are analysed by the ‘findpeaks’ function in Matlab. From the discovered frequencies, the two minimum frequencies are identified as characteristic frequencies. The significance of each characteristic frequency is tested by considering whether the value is independent of subject and floor conditions, using two-way ANOVA on subject and floor condition for this test.

### 4.6 Identification of model parameters

In the analysed model, the stationary state is stable when *k*_P0_>*mgh*. Cyclic motion appears when *k*_P0_ falls below *mgh*, so the existence of bifurcation can be analysed by identification of *k*_P0_. Identification of quiet standing without disturbance has been performed by the correlation between COM and EMG readings [14,18], linear regression [52], power spectrum [41], maximum-likelihood estimation [53] and other means. In the present research, estimation is performed by using the power spectrum because its information set is richer than that of the distribution. The parameters are searched so that the RMS error between the power spectrum of the measured data and that of the model obtained by simulation decreases. Searched parameters are the control gains *k*_P0_, *k*_P1_, *k*_P2_, *k*_D_ and *k*_I_, and the magnitude of noise *σ*. Response delay is set constant at 150 ms. Measured values for participants are used for body mass (*m*) and length from ankle to COM (*h*). The moment of inertia ( *J*) is calculated as *mh*^2^.

## Data accessibility

Motion data on fixed floor and rotational floors: Dryad http://dx.doi.org/10.5061/dryad.40j0j.

## Appendix A. Calculation of cyclic solution

The fundamental equation (4.5) is solved with the following conditions:

A 1 $$\left\{ \begin{array}{ll} \mathrm{solution}: & \theta =ae^{j\omega t}+a^{*}e^{-j\omega t}\\ \mathrm{restraint}\;\mathrm{condition}: & \dot{a}e^{j\omega t}+{\dot{a}}^{*}e^{-j\omega t}=0\\ \mathrm{approximation}\;\mathrm{of}\;\mathrm{integral}\;\mathrm{member}: & \int \theta dt=\frac{1}{j\omega }(ae^{j\omega t}-a^{*}e^{-j\omega t})=0. \end{array}\right.$$

By substituting equation (4.24) into equation (4.5), the following equation can be obtained:

A 2 $$\begin{array}{rl} jJ\omega (\dot{a}e^{j\omega t}-{\dot{a}}^{*}e^{-j\omega t}) & =-J{\left(\;j\omega \right)}^{2}(ae^{j\omega t}+a^{*}e^{-j\omega t})+(\text{mgh}-k_{P0})(ae^{j\omega t}+a^{*}e^{-j\omega t})\\ & \;-k_{D}j\omega (ae^{j\omega t}-a^{*}e^{-j\omega t})-\frac{k_{I}}{j\omega }(ae^{j\omega t}-a^{*}e^{-j\omega t})\\ & \;-k_{P2}(a^{3}e^{3j\omega t}+a^{*3}e^{-3j\omega t}+3a^{2}a^{*}e^{j\omega t}+3aa^{*2}e^{-j\omega t}). \end{array}$$

Here, the equation $\dot{a}e^{j\omega t}+{\dot{a}}^{*}e^{-j\omega t}=0$ is equivalent to ${\dot{a}}^{*}e^{-j\omega t}=\dot{a}e^{j\omega t}$, and by multiplying e^−*jωt*^ with both members, the above equation becomes

A 3 $$\begin{array}{rl} 2jJ\omega \dot{a} & =-J{\left(\;j\omega \right)}^{2}(a+a^{*}e^{-2j\omega t})+(\text{mgh}-k_{P0})(a+a^{*}e^{-2j\omega t})\\ & \;-k_{D}j\omega (a-a^{*}e^{-2j\omega t})-\frac{k_{I}}{j\omega }(a-a^{*}e^{-2j\omega t})\\ & \;-k_{P2}(a^{3}e^{2j\omega t}+a^{*3}e^{-4j\omega t}+3a^{2}a^{*}+3aa^{*2}e^{-2j\omega t}). \end{array}$$

By numerically integrating this equation for one cycle, the equation becomes

A 4 $$\dot{a}=-\frac{1}{2J\omega }\left(k_{D}\omega -\frac{k_{I}}{\omega }\right)a-\frac{j}{2J\omega }(\;J\omega^{2}+mgh-k_{P0}-3k_{P2}aa^{*})a.$$

## Appendix B. Calculation of variance of centre of mass

When the Langevin equation

$$\begin{array}{rl} \text{d}X & =AX\;\text{d}t+\text{d}W\\ X & =\left[\begin{array}{c} \eta \\ \theta \\ \psi \end{array}\right],\;A=\left[\begin{array}{ccc} 0 & 1 & 0\\ 0 & 0 & 1\\ -\frac{k_{I}}{J} & \frac{\text{mgh}-k_{P0}}{J} & -\frac{k_{D}}{J} \end{array}\right],\;\text{d}W=\left[\begin{array}{c} 0\\ 0\\ \frac{\sigma }{J}\xi \end{array}\right] \end{array}\},$$

correlation matrix *X* given by

A 5 $$R=\langle XX^{T}\rangle =\left[\begin{array}{ccc} \left\langle \eta^{2}\right\rangle & \left\langle \eta \theta \right\rangle & \left\langle \eta \psi \right\rangle \\ \left\langle \eta \theta \right\rangle & \left\langle \theta^{2}\right\rangle & \left\langle \theta \psi \right\rangle \\ \left\langle \eta \psi \right\rangle & \left\langle \theta \psi \right\rangle & \left\langle \psi^{2}\right\rangle \end{array}\right]$$

and the magnitude of noise

A 6 $$D=\langle \text{d}W\;\text{d}W^{T}\rangle =\left[\begin{array}{ccc} 0 & & \\ & 0 & \\ & & {\left(\frac{\sigma }{J}\right)}^{2} \end{array}\right]$$

have the relationship given by the fluctuation-dissipation theorem,

A 7 $$\text{AR}+{\text{RA}}^{T}=-2D,$$

then the members of *R* become

A 8 $$\left\{ \begin{array}{l} r_{12}=0\\ r_{23}=0\\ r_{13}=-r_{2}\\ r_{1}=\frac{k_{D}}{k_{I}}r_{2}\\ r_{3}=-\frac{mgh-k_{P}}{J}r_{2}\\ k_{I}r_{2}-k_{D}r_{3}=-\frac{\sigma^{2}}{J}, \end{array}\right.$$

and the variance of *θ* becomes

A 9 $$\langle \theta^{2}\rangle =r_{2}=\frac{\sigma^{2}}{k_{D}(k_{P}-\text{mgh})-Jk_{I}}.$$

Here, by substituting equation (4.15) into the equation of *θ*^2^

A 10 $$\langle \theta^{2}\rangle =\frac{\sigma^{2}}{k_{D}(k_{P0}+k_{P2}\langle \theta^{2}\rangle -mgh)-Jk_{I}},$$

which becomes

A 11 $$\langle \theta^{2}\rangle =\frac{1}{2k_{D}k_{P2}}\left\{Jk_{I}+{\text{mghk}}_{D}-k_{D}k_{P0}+\sqrt{{\left({\text{Jk}}_{I}+{\text{mghk}}_{D}-k_{D}k_{P0}\right)}^{2}+4k_{D}k_{P2}\sigma^{2}}\right\}.$$
